# Interhemispheric gravity‐assisted approach for microsurgical resection of a splenium arteriovenous malformation

**DOI:** 10.1002/ccr3.6853

**Published:** 2023-01-26

**Authors:** Jacob C. Harris, Austin J. Borja, Gregory Glauser, Donald K. E. Detchou, Susanna D. Howard, Omar A. Choudhri

**Affiliations:** ^1^ Department of Neurosurgery University of Pennsylvania Philadelphia Pennsylvania USA

**Keywords:** arteriovenous malformation, gravity retraction, microsurgical resection, splenial AVM

## Abstract

The parietal interhemispheric approach employing gravity retraction with skeletonization of bridging veins provides an excellent operative window for safe, curative resection of splenial arteriovenous malformations.

## Graphical abstract

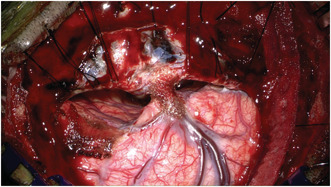

What are some operative techniques that can be employed for the safe, curative resection of splenial arteriovenous malformations? The parietal interhemispheric approach employing gravity retraction with skeletonization of bridging veins provides an excellent operative window for microsurgical resection of these lesion (Figure 1, Video S1).^1^

**FIGURE 1:**
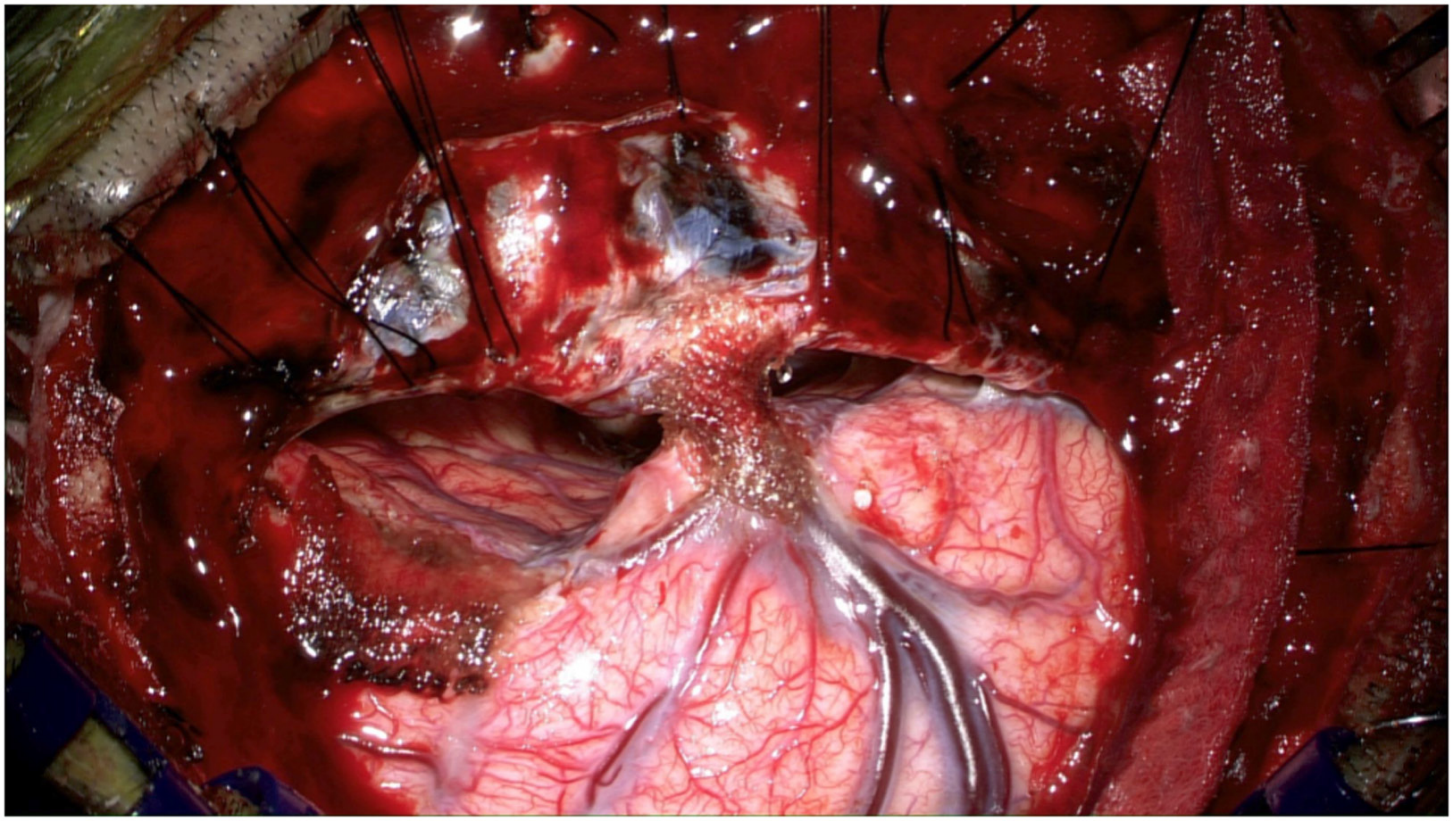
Operative window using the parietal interhemispheric approach.

## AUTHOR CONTRIBUTIONS

**Jacob C Harris:** Data curation; formal analysis; software; visualization; writing – original draft; writing – review and editing. **Austin J Borja:** Data curation; formal analysis; software; visualization; writing – original draft; writing – review and editing. **Gregory Glauser:** Software; writing – original draft; writing – review and editing. **Donald K E Detchou:** Writing – review and editing. **Susanna D Howard:** Conceptualization; investigation; writing – review and editing.

## CONFLICT OF INTEREST STATEMENT

The authors have no personal, financial, or institutional interest in any of the drugs, materials, or devices described in this video.

## ETHICAL APPROVAL

This video was deemed Institutional Review Board (IRB)‐exempt by the present institution's IRB as it is considered a case report, which does not require IRB approval. Strict adherence to all university, state, and federal requirements regarding patient confidentiality and care has been upheld.

## CONSENT

Written informed consent was obtained from the patient to publish this report in accordance with the journal's patient consent policy.

## Supporting information


Video S1.

## Data Availability

No data available.
